# Five Domains, Multiple Realities: A Multidimensional Analysis of Equine Welfare in Equestrian Events in Brazil

**DOI:** 10.3390/ani16182916

**Published:** 2026-09-16

**Authors:** Keity L. G. Trindade, Carolina J. F. L. Silva, Raíssa K. S. Cruz, José D. Ribeiro Filho, César F. Vilela, Vítor S. R. Vilela, Clarisse S. Coelho, Helena E. C. C. C. Manso, Helio C. Manso Filho

**Affiliations:** 1 Equine Research Center, Federal Rural University of Pernambuco (UFRPE), Recife 52171-900, PE, Brazil; 2 School of Veterinary Medicine, Cesmac University Center, Maceió 57051-160, AL, Brazil; 3 Department of Veterinary Medicine, Federal University of Viçosa, Viçosa 36570-900, MG, Brazil; 4 Independent Researcher, Americana 13474-470, SP, Brazil; 5 Veterinary and Animal Research Center (CECAV), Faculty of Veterinary Medicine, Lusofona University, 1749-024 Lisbon, Portugal

**Keywords:** horses, behaviour, sports, health, sustainability

## Abstract

Welfare assessments at traditional equestrian events in Brazil are scarce. This study aimed to systematize and stratify assessments based on the Five Domains (5DMs) model into subdomains (sDMs) for vaquejada and gait events and to adapt them to reflect local conditions and resource availability. Subsequently, the sDMs were classified and scored on a 5-level scale (1 to 5, where 1 = very poor and 5 = excellent), and assessments were conducted at three vaquejada and five gaited horse competitions. Assessments took place during morning, afternoon, and evening periods over two or more days and were carried out by previously trained evaluators. Overall, results indicated that the level of animal welfare was good or excellent across all five domains. Regarding the health domain, 2.8% of the animals were classified at “very poor” or “poor” levels due to a higher incidence of compromised sDMs. Analysis of the sDM results stratified by event revealed a higher frequency of scores below “fair” in the health domain (*n* = 12), followed by the environment (stalls) domain (*n* = 4) and nutrition domain (*n* = 2), a pattern reflected in the overall results. It is concluded that the use of the adapted 5DMs model and its subdomains (sDMs) demonstrates potential as a preliminary, exploratory framework for assessing the welfare of equine athletes at equestrian events, regardless of the event’s level or quality, regarding the impact of these events on the horses’ quality of life.

## Simple Summary

This study proposes a framework for assessing the welfare of horses at traditional Brazilian equestrian events (vaquejadas and gait competitions). The researchers utilized the “Five Domains” welfare assessment model, subdividing it into specific sub-domains to better characterize the horses’ quality of life during these events. Overall, horse welfare was rated as good or excellent across most domains, although certain sub-domains showed below-average or poor quality (health: 12 cases; environment: 4 cases; nutrition: 2 cases). Given the limited sample of events and the need to further define some sub-domains, the applied model serves as a proposal for assessing equine quality of life; therefore, future research should focus on better validating specific indicators to classify potential risks to welfare. Refining this process and implementing immediate preventive and corrective measures are expected to restore and maintain a positive level of welfare for the horses during events, while minimizing the risk of physical and mental suffering for the animals involved in competition.

## 1. Introduction

Scientific advances in animal welfare (AW) have been consolidated in recent decades as one of the fundamental pillars for the ethical and sustainable management of animals used in productive, recreational, and sports activities [1,2,3,4], incorporating the relationships among animals, humans, and the environment [5,6,7,8]. Thus, the well-being of all domestic animals clearly involves not only the absence of suffering but also the promotion of conditions that enable them to have a well-lived life and positive interactions with humans [6,7,9,10,11,12,13,14,15], resulting in mutual benefit to all and providing sustainability to this system in the environment.

Equids occupy a unique position among domesticated species, as they participate in a wide range of activities with humans, from agricultural work to high-performance competitive sports [16,17,18,19,20,21]. These interactions between equids and humans present specific challenges for maintaining adequate levels of animal welfare, such as routine feeding practices, isolated environments, and the training of young animals, which can currently be assessed through various metrics based on indicators and clinical signs, such as body condition score or composition, as well as heart rate recovery and variability, as described by researchers [1,12,13,18,19]. These metrics have incorporated innovations over time, including new assessment methodologies for various species [7,10,12,20]. Therefore, assessing animal welfare in equids is complex due to the diverse contexts of husbandry systems, management practices, and human–animal interactions worldwide [18,20,21,22,23,24,25,26,27,28,29,30,31,32,33,34,35].

Recently, researchers and society have promoted new models for assessing AW with the incorporation of new scientific and practical advances concerning AW in contemporary sports and other activities with horses [12,34]. However, equine sports activities currently face growing social demand for transparency, greater responsibility toward the animals on the part of those involved, and the sustainability of the equine industry regarding animal welfare practices and ethical treatment, in order to maintain their social license to operate (SLO) [3,13]. Animal welfare, when focused on the horses’ quality of life, is linked to the promotion of sustainability throughout the equine production chain. This fact underscores the importance of demonstrating—in equestrian competitions—the commitment of all those involved with horses through horse-centered welfare assessments, while acknowledging that this is a complex process grounded in scientific research [5,7,17,19].

In this context, the Five Domains Model (5DMs) and its subdomains (sDMs) [1,13], based on research into animal welfare assessment, emerged as a new set of conceptual and practical tools for this purpose. This system covers the DMs of environment, nutrition, health, behaviour, and mental state, and enables an integrated understanding of well-being that recognizes the interactions between the physical and affective dimensions of the animals’ experiences [1]. Furthermore, this approach using 5DMs and sDMs enables the implementation of real-time corrective measures, contributing to the continuous improvement of management and welfare conditions, such as the provision of forage, water, and a safe, shaded environment, while also facilitating the consolidation of good training and sporting practices. Thus, the application of the 5DMs and their sDMs aims to improve the horses’ quality of life in these activities, as adapting to the specificities of contemporary equestrian sports in the Americas represents a significant advancement for animal welfare assessments; this can economically consolidate these sports disciplines within their respective regions and countries while centering welfare on the horses’ quality of life.

Initially, the use of the 5DMs was formatted with a non-numeric scale so that it would not be transformed into simple mathematical results. However, a numerical scale could be used when they are only indications of a gradation of severity (positive, neutral, or negative), especially when the system is used longitudinally, with repetitions per day, and on a large scale. Thus, understanding and monitoring horse welfare through assessments during equestrian events can be essential not only for ensuring their quality of life but also for consistently maintaining a high standard of welfare and, potentially, reducing the risk of a decline in quality.

The lack of systematic studies applying up-to-date methodologies for animal-centered welfare assessment in the Americas represents a significant gap in both scientific and practical spheres, leading to the perception that there are no practices aimed at continuously maintaining animal welfare at these events [8,13,31]. Therefore, research and analysis of the AW situation in events can be used to quickly identify problems and improve positive aspects. Adapting and applying the 5 Domains Model (5DM) to contemporary equestrian disciplines, such as vaquejada and gait competitions, can provide novel insights into the actual welfare and quality of life of the animals involved. Furthermore, it can inform policies and recommendations aimed at promoting animal well-being through responsible practices that contribute to the sustainability of horse husbandry.

Based on this perspective, the present study was conducted to test the hypothesis that longitudinal assessments of horse welfare during equestrian events, using the Five Domains (5DMs) model and its respective sub-domains (sDMs), can effectively characterize the negative and positive aspects of animal welfare in traditional sporting disciplines. To this end, a preliminary program of longitudinal, systematic assessments based on the 5DMs, stratified into 79 sDMs, was established and applied to vaquejada [29,30] and gait competitions [27]. The objective was to present the preliminary framework of a practical, effective system for the continuous and systematic assessment of the welfare and quality of life of equine athletes in competition; such a system could facilitate the identification of potential welfare risks and enable real-time corrective actions, thereby strengthening the commitment of equestrian disciplines to the ethical, scientific, and legal principles underpinning the balanced coexistence of humans and animals, both now and in the future, in the interest of sustainability and the responsible use of equine athletes in competition.

## 2. Materials and Methods

### Application of the 5 Adapted Domains

Between 2023 and 2025, eight professional equestrian events were monitored across the disciplines of marcha (*n* = 5), which is a low-intensity, medium-duration (endurance) competition [27], and vaquejada (*n* = 3), which is a high-intensity, short-duration (strength) race [29,30] (Table 1), where the horses were evaluated by an adapted 5DM model. These events were public and used official competition rules according to the modality, with authorization from the official and health authorities. The environments of the events were available for public visitation between 05:00 and 23:00. The competitions took place between 8:00 am and 11:00 pm, with or without a break for lunch.

The proposed animal welfare assessment model was implemented by at least two assessors who had been trained beforehand and were familiar with the 5DMs and their respective sDMs. A pilot project was developed for this initiative, comprising a theoretical training phase to align perceptions and several practical sessions at events, during which the assessors observed the application of the assessment forms. The assessors were veterinarians and animal scientists holding at least a master’s degree and possessing over two years of experience in veterinary and animal welfare assessments at equestrian events.

**Figure 1:**
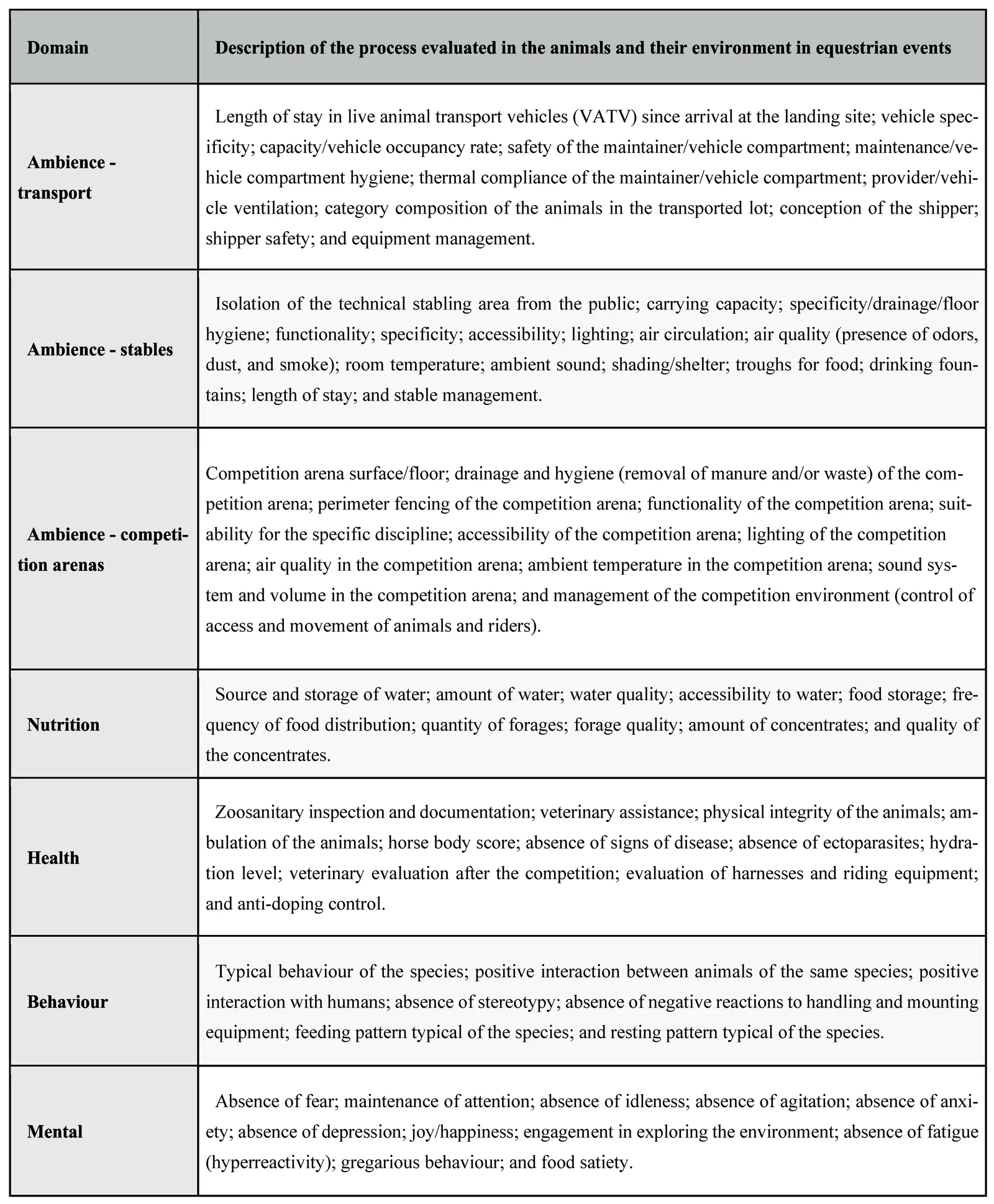
Individualized description of the domains and their subdomains used in the evaluations of horses present in monitored equestrian events.

Assessments took place following an initial brief, and the evaluators would then cross the horse stabling and competition areas randomly, yet ensuring coverage of all environments. In subsequent assessments, they could follow the same route or take a different one, provided the entire event venue was covered. During the assessments, the evaluators did not converse with one another. The electronic forms, which were developed from the 5DMs and their sDMs (Figure 1), were sent to the evaluators by email, accessed through mobile devices (cell phones and/or tablets), and filled out in real time in the morning (8 am–10 am), afternoon (2 pm–4 pm), and evening (7 pm–9 pm) on at least two days per event. Immediately after completion, the forms were sent to a server, where the results were stored for later analysis. Any situation indicating or potentially causing a reduction in animal welfare was reported to official veterinarians, in accordance with national legal and professional ethical standards, after the relevant forms were completed or, if necessary, in advance.

These electronic forms were developed according to different aspects of animal welfare described in the 5DMs, which are detailed in Figure 1, and distributed in the following themes:

- **Ambience (transport)**—equipment involved in the transport and loading/unloading of animals at the event site;
- **Ambience (stable environment)**—stables and all processes related to individual stabling (walking events) or group stabling, but with 4 or fewer horses from the same competition team (vaquejada);
- **Ambience (competition arena)**—arena/competition track and ancillary areas;
- **Nutrition**—all aspects of food at the venue;
- **Health**—different aspects of health from the entry and stay of the animals in the competition site until their exit, which included inspection of the animals and evaluations before, during, and after competitions;
- **Behavioural**—evaluation of the behavioural repertoire, with analysis of typical and atypical expressions of the horses present at the event site in any environment during the time period of the event;
- **Mental**—different aspects of the mental DM (positive and negative) of the horses present at the event site in any environment.

**Figure 2:**
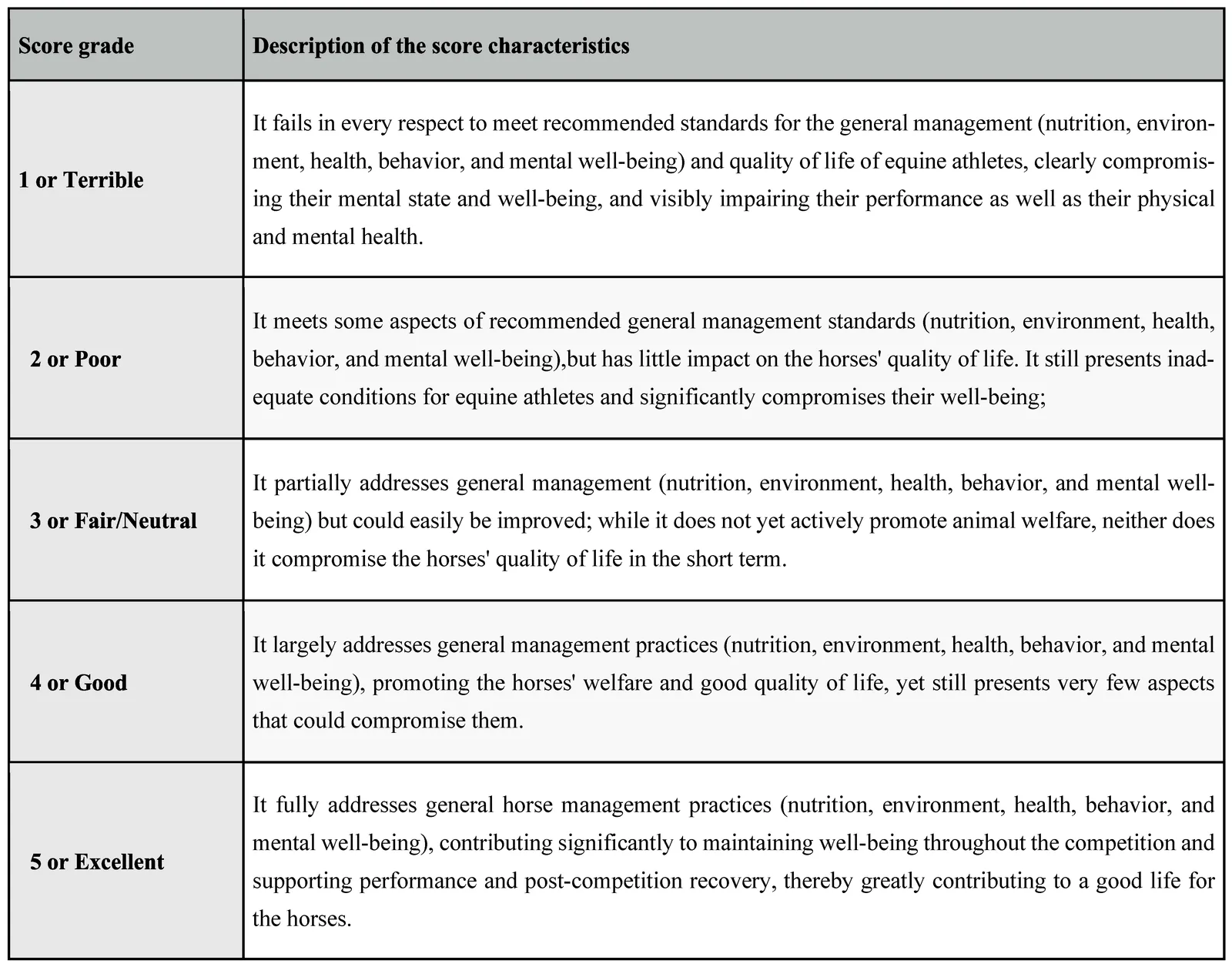
Description of the score characteristics for the classification of the 5DM and sDM during event evaluations.

At the end of the evaluations, the results available on the server were exported to spreadsheets of the Numbers app (Apple Co., Cupertino, CA, USA). For multidimensional understanding, regardless of sport, the means of the 5DMs and sDMs of each sport were added and divided by the total number of events. The results were subsequently transformed into scores from 1 to 5, which represent 1 (poor), 2 (poor), 3 (fair), 4 (good), and 5 (excellent) (Figure 2). With this numbering, the arithmetic means of each DM and sDM were obtained. The means of each sDM for all events were also calculated individually.

Finally, based on the numerical scores, the results of each DM and sDM were grouped into three groups (poor/very poor: means ≤ 3.0; regular: means ≥ 3.01 and ≤3.99; and good/excellent: means ≥ 4.0), and then the frequencies were calculated for an estimate of the possible risks in each DM and sDM, where poor/very poor results indicated high risk, fair indicated medium risk and good/excellent indicated a low risk of reduction in the quality of AW.

## 3. Results

### 3.1. General Results of the Evaluations

The combined results of this proposed study indicated a predominance of conditions classified as good/excellent across most domains of the adapted Five-Domain Model (5DM): environment [transport (83.0%), stalls (80.4%), and arena (94.2%)], nutrition (80.5%), health (67.6%), behavior (96.3%), and mental state (100.0%) (Table 2), suggesting that the management protocols and the implementation of practices observed at the evaluated events largely met animal welfare requirements and good management practices.

However, it should be noted that all results must be analyzed by taking into account the complex interactions between all domains and the professional experience of the evaluators in order to understand the quality of life of competition horses. These detailed analyses reveal specific weaknesses requiring greater attention, which could compromise the animals’ quality of life during and after the events, as well as positive aspects that should continue to be monitored to ensure the maintenance of the beneficial impact on the horses’ quality of life.

Furthermore, the low welfare levels observed in this study regarding specific welfare domains may have influenced the overall welfare quality results across those domains (environment [facilities: 2.7%], nutrition [2.2%], and health [15.0%]) by compromising the horses’ natural behavior; for instance, small or cramped facilities can reduce resting space, restricted access to water can lead to varying degrees of dehydration, and health issues, such as pain in the musculoskeletal system or at the bit contact point, can affect the horses. Horses that do not rest or are in pain likely experience compromised mental well-being, becoming more reactive and potentially altering their behavior toward riders.

Finally, results for the combined 5DMs, regardless of event location or the specific sDMs, indicated that the analyzed events exhibited varying levels of welfare quality: very poor/poor (2.8%), fair (11.1%), and good/excellent (86.0%). This finding demonstrated that there was still room for well-being to be compromised, even though these aspects could be improved; the negative points were clearly identified and, despite their low frequency, could impact the horses’ quality of life throughout the competition.

### 3.2. Ambience

Due to the complexity of the ambience DM, in the current study, this domain was divided into three groups (transport (Table 3), stables (Table 4), and competition arenas (Table 5)) for more specific evaluation in athletic horses. The results of the combined evaluations of the three divisions of the ambience DM, regardless of the event, indicated that the evaluations presented mostly good/excellent levels of quality of well-being, which was expected because they were official events, regulated, and had practices already established with competent authorities. The quality of the level of well-being identified in the ambience DM, which associates adequate infrastructure with lower levels of stress and adverse physiological responses, has been studied under various conditions [6,14,20,25,28].

When the ambience DM with its three macro divisions (transportation, stables, and competition arenas) (Table 3, Table 4 and Table 5) and its sDMs, was analyzed, the level of quality of well-being of the transportation was found to be predominantly good/excellent (83.0%), with the remaining portion classified as regular (17.0%), principally associated with the loading and undloding area. Regarding the stables, the ratings were good/excellent (80.4%), fair (16.8%), and poor/very poor (2.7%); this aspect of the environment showed the highest degree of compromise across various factors, with the lowest scores observed in three events: one vaquejada and two gait competitions. Finally, when the events were analyzed separately, only one of them presented fair results for all three macro divisions of the ambience DM, and in the other seven, the results were all good/excellent.

### 3.3. Nutrition

Similarly to what was observed in the evaluation of the ambience DM, the evaluations of the nutrition DM and its sDMs were able to identify a reduction in the quality of well-being in different situations (Table 6). An analysis of the independent overall means of the events revealed that all sDMs had means above or equal to 4.0 (good/excellent). However, when the event averages were evaluated individually, two walking events showed averages classified as fair (>3 and <4) and were associated with problems regarding the regular supply of water to the animals.

The frequency of the average quality of well-being of the nutrition DM was 80.5% for good/excellent, 17.3% for fair, and 2.2% for poor/terrible. It was also observed that in the two sDMs of nutrition (source/storage of water and amount of water), the mean frequency of the level of well-being was poor/terrible (12.0%); only one sDM of nutrition (food accessibility) showed a frequency of 100% for good/excellent.

### 3.4. Health

In terms of the health DM, an analysis of the overall means regardless of the events revealed that seven sDMs had means above 4.0, and two sDMs had means below 4 but above 3.0. Only one mean was less than 3.0 (veterinary evaluation after the competition). An analysis of the means of the sDMs per event revealed that in three events, the means were less than 4 but greater than 3.0. The overall mean of the events for the DM was 4.04 (Table 7).

The frequencies of the level of well-being analyzed for the health DM were 67.6% good/excellent, 17.5% fair, and 15.0% poor/terrible. An evaluation of the 10 sDMs revealed that four sDMs had poor/terrible welfare levels: two had values of 13.0% (animal health inspection and zoosanitary documentation), and two had values of 62.0% (veterinary evaluation after the competition and evaluation of driving and riding harnesses). In only three sDMs, the frequency of quality of well-being was 100.0% (animal ambulation, body score of horses, and absence of ectoparasites).

### 3.5. Behavioural and Mental

In the present study, the means of the sDMs were above 4.0, both in the independent analyses of the events and in the individual events (Table 8 and Table 9). The frequency of the level of well-being in the five sDMs was 100% good/excellent, and two had frequencies classified as regular. For the behaviour DM, the frequency of the well-being level was 96.3% good/excellent and 3.6% fair (interactions with animals of the same species and with humans). Despite the low frequency of regular results, these two sDMs can be easily improved with more appropriate training in horsemanship practices and with periodic recycling.

## 4. Discussion

### 4.1. General Comments on the Evaluations

Applying the proposed model, adapted from the 5DM and sDM frameworks, to equine athletes competing in gait and vaquejada events demonstrated its potential as a comprehensive, sensitive tool for the longitudinal, multidimensional assessment of animal welfare. This approach seeks to address the complexities of assessing animal welfare in equestrian competitions and reinforces the importance of methods that integrate multiple environmental, physiological, and behavioral dimensions, as recommended and refined in updated versions of the 5DM model and other scientifically and practically grounded systems [7,8,10,20].

The system proposed here, which is an adaptation of the 5DM and sDM models used in this study, combined with a risk assessment to determine the frequency of welfare quality levels, proved to be a methodology that contributes to identifying negative points requiring rapid correction and positive points that should be reinforced. Thus, the application of this longitudinal and multidimensional assessment proposal provided further details regarding the negative or positive conditions affecting horses involved in equestrian disciplines in the Americas.

Several studies on welfare, particularly regarding equine athletes, have recently been published, reporting varying perceptions among the general public and those within the horse industry regarding the quality of life of equines, as well as the quality and implications of their welfare [22,23,31,36]. For example, the study by the Equine Ethics & Wellbeing Commission of the International Equestrian Federation [23] identified some deficiencies in different domains in the interviews. In the nutrition DM, 11% of the respondents reported that horses received little forage or inadequate diets. In the ambience DM, 24% of the respondents indicated a reduction in free time or small boxes, and in the behavior DM, 39% of the respondents reported a reduction in time for socializing the animals and limited understanding of the basic needs of the horses.

Additionally, in this FEI study [23], weaknesses were detected in the health DM, as concerns with animal inspection and lack of frequent veterinary checks were indicated, in addition to the misuse of driving and riding harnesses. Both the results of the FEI [23] study and those of other analyses [7,8] are consistent with the results obtained in this current study. Recently, it was described that in addition to a good quality of life, horses need a good environment, socialization, and access to forage [14]. These findings indicate that both society and researchers have sought to improve the general welfare conditions of equines around the world.

### 4.2. Ambience

Analysis of all 39 sDMs of the ambience DM revealed that welfare quality levels were good/excellent or regular in 35 sDMs and poor/very poor in 4 of the sDMs, which were in the stables in three different events (Table 4). Furthermore, when the sDMs of the ambience DM (stables) were analysed, poor/terrible results were observed in 2.7% of the cases for this DM and occasionally in four sDMs (floor hygiene, poor shading, problems in the water troughs of the boxes, and isolation of the public from the stables).

Poorly maintained stalls foster the onset of conditions such as hoof ailments and respiratory diseases, which can rapidly compromise the health domain. This is particularly concerning when hygiene issues coincide with other deficiencies, such as a lack of shade and faulty waterers, that can impact the hydration levels of the equine athletes. However, at least three of these four sDMs can be quickly rectified once identified; in contrast, the issue of isolation from the public must be addressed prior to the event through temporary or permanent structural measures. Isolating the animals from the public ensures their safety and allows for rest, while also protecting spectators, who may be unfamiliar with horses, thereby preventing accidents.

**Figure 3:**
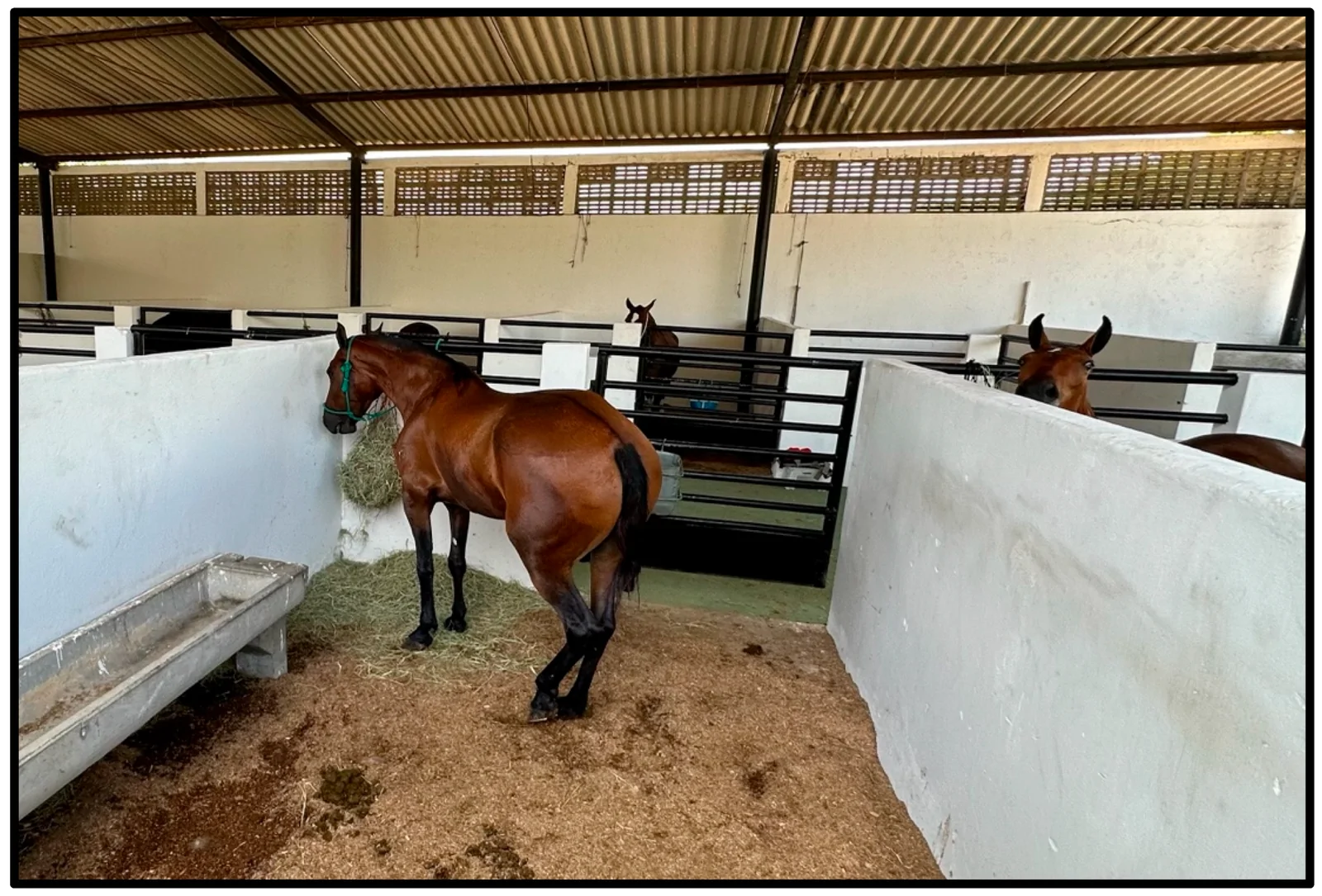
The typical stable setup for horses in gaited horse competitions involves the animals being kept in stalls with a wide view of each other and access to water and forage throughout the day.

**Figure 4:**
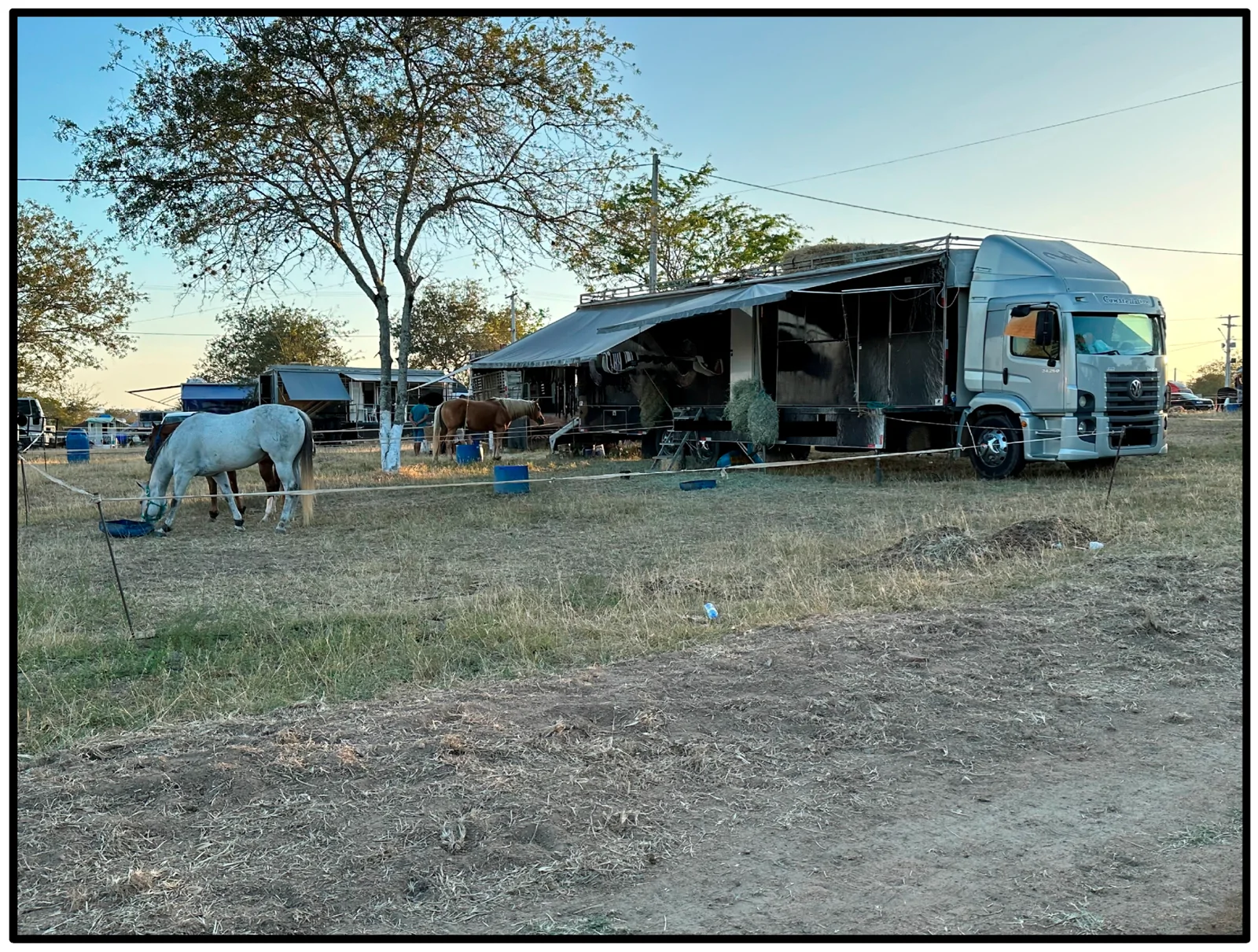
A typical stable layout for horses used in *vaquejada* events, where the animals are kept in small, open areas with access to water, forage, and the handlers’ rooms throughout the event.

The ambience DM can also be affected by the conditions of the local biome and the needs of each equestrian sport, and adapting older sites to welfare practices and current legislation is always difficult. In this sense, the places where the vaquejadas and the gait events are performed are quite different, and the way the horses are stabled is the greatest difference, which can affect the behavioural and mental DMs. During the vaquejadas, the horses are regularly housed collectively, which favours horses’ socialization, and can still walk freely and interact socially, in addition to having free access to water and forage throughout the day (Figure 3). In contrast, in the gait events, the horses were housed in individual boxes but with ample view of the other horses in the event, allowing physical, auditory, and olfactory contact, free movement, and regular access to forage and water (Figure 4).

Another important aspect of both systems mentioned above is that the grooms were housed alongside the horses under their care. During the day, all activities, such as grooming and cleaning the stall bedding, were carried out within the stalls and were accompanied by playful interactions between the horse and the groom. At night, the grooms slept in hammocks in the stable aisle (Figure 3), and horses were observed resting or sleeping beside them, albeit from within the stall. In the case of the group housing model (Figure 4), animal and human behaviors were very similar to those described for the individual stalls. This model of frequent human-horse interaction optimizes the human-horse relationship in both sporting disciplines, contributing to positive behavioral and mental outcomes for the animals [6,7] and fostering behaviors and interactions with the grooms that enhance the horses’ quality of life and positive welfare [3,32,33].

These two types of structures mentioned above tend to promote the natural behaviours of the species, which relate to other forms of enrichment, such as social, sensory, and occupational enrichments, leading to the promotion of positive interactions and strengthening skills such as adaptation to new conditions and affective processes, which increase well-being [7,20]. It was recently shown that athletic horses, when accommodated in groups, have greater social interaction and facilitate free exercise [25]. Compared with completely closed boxes, boxes with greater interactions (visual and physical) and free access to food (fodder and water) can also result in more social interactions [7,14,34], in addition to providing greater safety to stabled animals. The benefits of these types of housing are reflected by the cortisol levels, similar to those of animals on breeding farms observed by *Aurich* [35]; however, studies are needed to evaluate different stables in equestrian events in Brazil from traditional sports (horse racing, show jumping, etc.)

Finally, these results regarding the environmental dimension suggest that locations with greater public exposure, such as transport areas and access routes to competition arenas, require heightened care for the animals, as horses, people, and vehicles share the same space. These transport and access areas also involve monitoring animal welfare, health requirements, and animal identification. In contrast, when the horses were housed in their stalls, the level of concern regarding their behavior among grooms and handlers appeared to decrease, as the animals were safe and had access to water and forage. This is most likely due to the intense focus on the competition itself. Therefore, when assessing animal welfare at equestrian events, all aspects of the environmental dimension should be monitored with equal intensity—for instance, through regular checks like those conducted in the present study, involving multiple visits over several days and at different times (morning, afternoon, and night).

### 4.3. Nutrition

Nutrition, which includes water and food, should be analyzed to always characterize the level of welfare of horses and meet the physiological characteristics of horses [8,13]. Some studies have shown that feeding programs do not yet rank among the primary concerns of those who manage horses [8,15,18,20,37], particularly regarding the availability of water and forage over 24 h. However, when horses have limited access to water or forage and remain confined for extended periods with restricted movement, they may exhibit signs of discomfort and pain, such as those associated with colic syndrome [3,7,35]. However, subtle signs of pain may go unnoticed by poorly trained evaluators; nevertheless, the analysis of other signs, such as reduced mobility or apathetic interactions with grooms and riders, can aid in diagnosis, especially when evaluations are repeated at different times or on different days, as was done in this study.

Thus, providing diets that are suited to the horses’ athletic activity, delivered regularly, and tailored to individual needs is expected to contribute to the quality of life of equine athletes [8]. Both athletic and non-athletic horses benefit from increased access to forage, regarding both health maintenance and natural behavior [38,39]. Feeding programs for athletic horses vary considerably even within the same groups. They range from the use of fresh or hay-based forage and concentrates rich in non-structural carbohydrates, or in oil and fiber, to homemade concentrate formulations. Therefore, greater attention should be paid to feeding programs during events, including individualized diets that meet the nutritional requirements of specific equestrian disciplines (whether endurance or speed-based) and ensuring free access to water and forage.

Drinking water must be available regularly, 24 h a day, during events; however, in many cases, this availability depends on constant monitoring of water troughs to check water levels and quality. This issue had previously been addressed in the context of water supply problems in the stalls. Interestingly, in the present study, factors related to the environment and nutrition were found to be associated with water availability, an unexpected result, given that athletic horses, especially those participating in endurance events or performing repetitive efforts at short intervals, require large amounts of water to replenish losses resulting from physical exertion.

The supply of water to athletic horses should be a concern in equestrian sports because sweat loss can be high because of the duration of competitions, specific aspects of stabling, and climatic conditions [7,40]. The reduction in the quality of well-being in the sDMs related to water and forage may be linked to the types of water troughs and troughs used in vaquejada events and gait events. At these events, the water troughs and feed buckets are removable, and the horses often play with them, especially when they are empty or contain little water or feed. This requires the grooms to pay close attention to keeping the troughs filled with water and the hay basket throughout the event, a task made easier by the fact that they remain with their animals 24 h a day.

Concerning forage in the evaluated events, the athletic horses received hayed forage of good quality regularly throughout the day. This process was facilitated by the constant presence of grooms, who were housed close to the horses as described above. Even though the amount of forage was placed as a critical point in different evaluations [7,13,23], in the current study, the nutrition DM was positive, with the forage received being of good quality and readily available, in addition to concentrate that was offered according to the sport category in the three analyzed periods.

### 4.4. Health

Comparatively, recent studies that applied the 5DMs model in horses also reported a certain degree of intra- and interdomain variability [4,13,41], especially in the dimensions related to health and nutrition. These findings corroborate the approach applied in the current experiment by seeking to analyze in detail the health DMs with their sDMs in vaquejada and gait horses to find critical points that need to be better monitored, especially in pre- and post-competition inspections, for the detection of horses that are not “fit to compete” and animals that were injured in the races or show signs of fatigue.

In this sense, one of the most important aspects for athletic horses, regardless of equestrian discipline, is the characterization of fatigue as a cause of distress. Fatigue is easily identified using various methods, like elevated heart rate during recovery and signs of dehydration, such as prolonged skin turgor time [42,43,44]. In competitions, the most widely used method has been the determination of post-exercise or recovery heart rate, with evaluations in up to 30 min, which has a scientific basis and can be easily checked by veterinarians [44]. The recovery heart rate, when combined with other assessments, such as examination of lameness and degree of hydration, can clarify those involved if they have the “fitness to compete”, which defines the situation of horses in competitions.

In competitions such as vaquejada and gaited events, recovery heart rate is not evaluated in the post-exercise phase, even though it has been shown under competition conditions that the horses in these sports, when well trained and nourished, recover well in up to 30 min after exercise. However, heart rate and hydration assessments may be conducted at any point the welfare officials deem necessary. In the current study, most of the negative evaluations of the health DM were related to carelessness in the evaluations of horses before and after competitions, a problem that had already been addressed in different studies [3,13,21,23], especially evaluations of the horses after the competition and the effect of driving and riding harnesses, in addition to physical distress. The latter could be quickly identified by evaluating the recovery heart rate, as previously mentioned.

The events sponsored by the FEI and the International Federation of Equestrian Authorities (IFHA) had well-defined rules regarding pre- and post-competition evaluations for a long time and have undergone regular improvements, unlike contemporary equestrian disciplines. Additionally, in the analyses of the *Delphi* study [13], there are indications that regular evaluations of different aspects of the health DM are important for the welfare of athletic horses and that society has paid attention to these aspects.

Currently, in Brazil, only the vaquejada and the rodeos are the events with the most current competition and welfare rules approved by the official control agencies, which include horses and cattle. However, several other equestrian modalities involving equines, with or without cattle, still require better regulation and/or are in the process of being made official by the official authorities. There is also no indication of broad programs for the analysis of well-being in these competitions, but we hope that this current study can contribute to a faster evolution of well-being evaluation systems with a scientific basis for the development of these programs, which are logically adapted to the different equestrian disciplines according to needs and particularities.

In terms of the health DM, the official rules do not indicate the obligation of anti-doping measures, and no type of official control for the use of drugs in the competitions was evaluated in this study. An FEI study [23] also revealed that there is concern about the indiscriminate application of drugs, especially those that mask health problems or are used for doping. In Brazil, the sale and application of medicines for horses are free, and essentially, anyone can purchase numerous types of products, which makes it difficult to control medicines in different contemporary and traditional sports. Finally, it should be noted that, without regulation for the use of drugs or without adequate control, this indiscriminate use may compromise human health and the environment. The maintenance of animal welfare and good competition practices should also meet the concepts of “One Health” and the sustainability practices of the horse production chain.

### 4.5. Behavioural and Mental

The behavioral domain, which is linked to animal physiology, has sparked numerous discussions within the equestrian community, stemming both from a lack of knowledge regarding the behavior of domestic horses, whether in free-ranging environments or on farms, and from differences in the observers’ perceptions and technical backgrounds. Debates on equine behavior often focus on the long-term impacts of domestication over the 5000 years since the process first began [37,45]. This process favored human needs (an anthropocentric approach), resulting in modifications to morphology, physiology, neurology, and behavior [19,37,45], changes similar to those observed in other domesticated species [46,47].

These evolutionary changes, such as increased docility and a reduced pain response, did not completely compromise the species’ biological needs, such as the continuous search for food, regular rest, and social interaction; this holds even though, compared to wild equids, there are modifications in stamina and gut microbiota necessitated by adaptation to human requirements [37,45,46,47]. More recently, some authors have demonstrated the impact of diet on horse behavior, linking it to the adaptation of the microbiota to the nutritional programs of athletic horses and equine performance [48,49], which need more studies to improve our comprehension of these processes.

Therefore, given the complexity of the subject, behavioral assessments of equids must avoid equating an animal’s environmental tolerance with the attainment of positive welfare. Such assessments must take into account behavioral modifications shaped by domestication while remaining grounded in the horse’s biological requirements. This approach should be facilitated by applying the objectives of the 5DM framework and its subdomains. Maintaining a good quality of life for the horse through frequent, regular assessments using the 5DM and its subdomains during competitions can sustain positive animal welfare at equestrian events by identifying risks to quality of life and preventing any decline in welfare [19,37,45,46].

In both types of events analyzed, horses were housed in groups belonging to the same team, whether collectively or individually, in stabling systems that allowed the animals to exhibit their typical behaviors, even when housed individually. The vaquejada horses benefit from group housing; in this setting, animals were observed displaying species-specific behaviors, such as rolling in the sand, maintaining physical contact, and resting in groups, that are not seen in animals kept in individual stalls without visual contact with others. In the gait competitions, the horses were housed in individual stalls but had a clear view of other horses and easy access for physical contact. These stabling characteristics in the evaluated events differ significantly from those observed in horses housed at jockey clubs and equestrian centers for show jumping, where horses spend much of the day confined and isolated from other horses. It is well known that confinement in fully enclosed stalls for prolonged periods can cause various forms of distress [31,32,33,34,35,38]; such conditions should be avoided to prevent compromising the horses’ behavior and mental state.

The mental DM is linked to the psyche of the animals and presents some difficulties in its evaluation, especially when the animal is unknown and/or the evaluator has temporary contact with it during the scans of the horses in the events. Subtle changes in the quality of the mental DM may be difficult to detect under practical conditions; hence, the presence of the groom and the riders involved with the horses in the event is important.

The predominance of good classifications in the mental DM is in line with the literature that highlights the importance of psychological adaptation and positive horse–human interactions [6,20], which occur regularly in the evaluated competitions. A potential lack of communication or positive interaction between horse and human can alter the animal’s mental state and lead to a refusal to obey the handler’s or rider’s commands [24,26], thereby compromising mental well-being. Conversely, the presence of handlers fosters mental well-being, as they meet basic needs and constantly assess and rectify any deficiencies [7,32,33]; when this is linked to other positive domains, a mental state with minimal impairment becomes evident. These procedures also help those involved with the animals to quickly recognize physical and mental distress and to take measures so that the distress does not progress to chronicity and does not compromise the welfare of the horses in these competitions.

Assessment of the behaviour and mental state of horses is laborious and time-consuming [36,50]. However, it can be argued that the results revealed, especially for the behavioural and mental DMs and their sDMs, certain moments associated with the DMs throughout the animals’ evaluation period. We also recognize that the limited number of events evaluated (*n* = 8) and the difficulty of conducting longitudinal evaluations represent important methodological restrictions, in addition to the high operational cost of the detailed application of the 5DMs and their sDMs. However, the adoption of new technologies for evaluating the welfare of athletic horses can reduce these difficulties [30,36].

Furthermore, from a practical standpoint, the preliminary results of all these evaluations, conducted during vaquejadas and gait competitions in Brazil, reinforce the future potential of the 5DMs system and its sDMs as a practical, efficient tool for the continuous monitoring of equine athlete welfare. This enables the early identification and correction of risk situations, provided that a good description of the mental domain is incorporated. Various authors have noted that recognizing issues and solutions regarding equine welfare helps preserve the “social license to operate,” thereby contributing to the consolidation of ethical and sustainable practices across the equestrian sector’s production and sports supply chains [2,8,11].

Finally, we acknowledge that point-in-time assessments during competitions may not reflect the reality over a full 24-h period, given the complexity of welfare analysis and the myriad interactions among the components of the Five Domains Model (5DM). However, since the assessments were conducted over two or more days at various times (morning, afternoon, and evening) by multiple assessors, the current results are significant; they offer a stratified overview of the horses’ experiences during these events, highlighting aspects that could be improved through remote assessment systems. Thus, the methodology proposed in this initial project, which is structuring an animal welfare assessment system based on the 5DM framework, could help transform practices that compromise horses’ quality of life and reinforce appropriate management and welfare standards. This initial project is expected to lay the groundwork for future studies that broaden the scope of sampling to include other disciplines and types of equines (such as donkeys and mules) and incorporate additional non-invasive techniques, such as thermography, video monitoring, and various AI applications, to enhance the sensitivity and applicability of these assessments, without ever excluding the human capacity to interpret equine behavior with clarity and sensitivity.

## 5. Conclusions

The use of the adapted Five Domains Model (5DM) and its sub-domains (sDMs) shows potential as an exploratory tool and a preliminary framework for monitoring the welfare of equine athletes at equestrian events—regardless of the event’s level or nature—thereby potentially improving the animals’ quality of life. This approach can assist in identifying situations where animal welfare may be compromised and indicates potential risk levels, while also allowing for regular updates or adaptations. Serial assessments, conducted at different times of day and over extended periods, also contribute to a more comprehensive understanding of the complex factors influencing animal welfare quality in contemporary competitive contexts, such as vaquejadas and gait competitions.

However, it is crucial to highlight that the current framework proposed for assessing equine welfare in competition horses, utilizing the Five Domains Model (5DMs) supplemented by specific domains (sDMs), may serve as an initial step toward better understanding the interactions between different aspects of equine welfare. Acknowledging the complexity involved, this initial proposal still requires the incorporation of new data, testing across other equestrian sports, and refinements to the methodologies used to aggregate the assessed domains. Consequently, future research should focus on validating specific indicators and clinical signs to facilitate the identification of risks to equine quality of life. Such validation is essential to enable the routine, large-scale application of this framework at equestrian events, aiming to implement preventive and corrective measures that ensure the recovery and maintenance of positive equine welfare while minimizing the risk of physical and mental suffering.:

## Figures and Tables

**Table 1:** Characteristics of the equestrian events monitored during the current study.

Event	Total Number of Evaluations	Duration of the Event, Days	Sport	Number of Horses *	Stabling Model	Breeds	Age Group
**Vaquejada**	8	4	Vaquejada	850	Collective	MQ	Youth, adults
**4-beat tests**	8	3	Gaited horse	90	Individual	MM, CAMP	Youth, adults
**Vaquejada**	25	5	Vaquejada	1100	Collective	MQ	Youth, adults
**4-beat tests**	35	5	Gaited horse	270	Individual	MM, CAMP	Youth, adults
**4-beat tests**	33	5	Gaited horse	290	Individual	MM, CAMP	Youth, adults
**4-beat tests**	32	4	Gaited horse	110	Individual	MM, CAMP	Youth, adults
**4-beat tests**	16	4	Gaited horse	277	Individual	MM, CAMP	Youth, adults
**Vaquejada**	18	5	Vaquejada	1100	Collective	MQ	Youth, adults

**Notes**: CAMP—Campolina breed; MM—Mangalarga Marchador breed; QM—Quarter Horse breed; young athletic horses (more than 3 years of age and less than 5 years of age) and adults (over 5 years of age and under 20 years of age); *: number of animals that circulated during the events.

**Table 2:** General results of the evaluations of the 5 domains and their sDMs according to the events (**A**) and their risks and levels of quality of well-being (**B**).

**5 Domains**								
(**A**) **General evaluations of the events according to the different domains**								
**Events**	**According to the 5 domains and their sDMs**							**Overall means of the 5DMs per event**
	**Ambience (transport)**	**Ambience (stables)**	**Ambience (arenas)**	**Nutrition**	**Health**	**Behaviour**	**Mental**	
**Vaquejada (*n* = 8)**	4.6	4.3	4.7	4.5	4.3	4.7	4.5	**4.5**
**Gait (*n* = 8)**	4.3	4.5	4.7	4.2	4.5	4.5	4.4	**4.4**
**Vaquejada (*n* = 25)**	4.1	4.3	4.2	4.2	3.7	4.5	4.5	**4.2**
**Gait (n = 35)**	4.6	4.4	4.7	4.8	4.2	4.8	4.8	**4.6**
**Gait (*n* = 33)**	4.8	4.8	4.9	4.9	4.3	4.9	4.9	**4.8**
**Gait (*n* = 32)**	3.9	3.8	3.9	3.6	4.2	4.1	4.3	**4.0**
**Gait (*n* = 16)**	4.2	4.1	4.4	3.9	3.2	4.3	4.4	**4.1**
**Vaquejada (*n* = 18)**	4.3	4.4	4.5	4.4	3.8	4.7	4.7	**4.4**
**Means of subdomain evaluations**	**4.3**	**4.3**	**4.5**	**4.3**	**4.0**	**4.6**	**4.6**	
**Overall mean of the evaluations of the 5 domains**								**4.4**
(**B**) **General frequency of the levels of quality of well-being and risks according to the 5 domains**								
**Quality levels of AW and risks**	**Mean frequency of the DMs and their sDMs**							**Mean frequency of the 5DM levels**
	**Ambience (transport)**	**Ambience (stables)**	**Ambience (arenas)**	**Nutrition**	**Health**	**Behaviour**	**Mental**	
**Good/excellent** **(>4.0);** **low risk**	83.0%	80.4%	94.2%	80.5%	67.6%	96.3%	100.0%	**86.0%**
**Fair** **(3.01–3.99);** **medium risk**	17.0%	16.8%	5.8%	17.3%	17.5%	3.6%	0.0%	**11.1%**
**Terrible/poor** **(<3.0);** **high risk**	0.0%	2.7%	0.0%	2.2%	15.0%	0.0%	0.0%	**2.8%**

**Notes**: *n* represents the number of evaluations per event. The scoring scale ranged from 1 (poor) to 5 (excellent) (Figure 2); DM: domain; AW: animal welfare.

**Table 3:** Mean results of the evaluations of the ambience domain-transport and its subdomains (*n* = 11) according to the events (**A**) and their levels of quality of well-being (**B**).

	**Ambience Domain—Transport**											
(**A**) **Evaluations of the events according to the different sDMs of ambience—transport**												
**Events and number of evaluations**	**sDM (transport) of the ambience DM**											**Overall means of the sDMs per event**
	**A**	**B**	**C**	**D**	**E**	**F**	**G**	**H**	**I**	**J**	**K**	
**Vaquejada (*n* = 8)**	5.0	5.0	4.9	4.9	4.5	4.6	4.9	4.9	3.0	3.8	4.6	**4.6**
**Gait (*n* = 8)**	4.4	4.4	4.5	4.3	4.3	4.1	4.4	4.5	4.0	4.0	4.1	**4.3**
**Vaquejada (*n* = 25)**	4.1	4.1	4.1	4.1	4.1	4.1	4.1	4.1	4.2	4.2	3.8	**4.1**
**Gait (*n* = 35)**	4.3	4.9	4.8	4.7	4.9	4.7	4.9	4.9	3.7	3.6	4.6	**4.6**
**Gait (*n* = 33)**	4.5	5.0	4.7	5.0	5.0	5.0	5.0	4.9	4.3	4.3	5.0	**4.8**
**Gait (*n* = 32)**	3.9	4.0	4.0	4.0	3.9	3.8	3.9	4.0	3.9	3.9	3.7	**3.9**
**Gait (*n* = 16)**	4.4	4.4	4.4	4.4	4.4	3.8	3.8	4.1	4.3	4.1	4.1	**4.2**
**Vaquejada (*n* = 18)**	4.4	4.5	4.4	4.4	4.4	4.4	3.4	4.4	4.4	4.3	4.2	**4.3**
**Mean of the evaluations of the subdomains**	**4.4**	**4.5**	**4.5**	**4.5**	**4.4**	**4.3**	**4.3**	**4.5**	**4.0**	**4.0**	**4.3**	
**Overall mean of the ambience domain-transport**												**4.3**
(**B**) **General frequency of the levels of quality of well-being and risks according to the sDMs of the ambience DM-transport**												
**Quality levels of AW and risks**	**sDM (transport) of the ambience DM**											**Ambience DM** -**transport**
	**A**	**B**	**C**	**D**	**E**	**F**	**G**	**H**	**I**	**J**	**K**	
**Good/excellent** **(>4.0);** **low risk**	87%	100%	100%	100%	87%	75%	63%	100%	63%	63%	75%	**83.0%**
**Fair** **(3.01–3.99);** **medium risk**	13%	0%	0%	0%	13%	25%	37%	0%	37%	37%	25%	**17.0%**
**Terrible/poor** **(<3.0);** **high risk**	0%	0%	0%	0%	0%	0%	0%	0%	0%	0%	0%	**0.0%**

**Notes**: A—length of stay in the vehicle since arrival at the disembarkation site; B—vehicle specificity; C—capacity/stocking rate; D—safety of the cargo compartment; E—hygiene of the cargo compartment; F—thermal conformance of the cargo compartment; G—cargo compartment ventilation; H—category composition of the animals in the transported lot; I—shipper’s conception; J—safety of the shipper; K—equipment management. *n* represents the number of evaluations per event. The scoring scale ranged from 1 (poor) to 5 (excellent) (Figure 2); sDMs: subdomains; DM: domain; AW: animal welfare.

**Table 4:** Mean results of the evaluations of the ambience domain—stables and its subdomains (*n* = 18) according to the events (**A**) and their levels of quality of well-being (**B**).

	**Ambience Domain—Stables**																		
(**A**) **Evaluations of the events according to the different sDMs of ambience—stables**																			
**Events and number of evaluations**	**sDM (stables) of the ambience DM**																		**Overall means of the sDMs per event**
	**A**	**B**	**C**	**D**	**E**	**F**	**G**	**H**	**I**	**J**	**K**	**L**	**M**	**N**	**O**	**P**	**Q**	**R**	
**Vaquejada (*n* = 8)**	3.6	4.4	4.6	4.4	4.5	4.3	4.5	4.4	3.9	4.6	4.9	4.8	3.6	2.9	5.0	5.0	4.6	4.0	**4.3**
**Gait (*n* = 8)**	4.4	4.6	4.1	3.8	4.8	4.8	4.6	4.8	4.6	4.6	4.6	4.4	4.6	4.9	4.5	4.4	4.4	4.1	**4.5**
**Vaquejada (*n* = 25)**	4.1	4.3	4.1	4.4	3.9	4.1	4.1	4.0	4.4	**4.5**	4.6	4.4	4.0	4.3	4.2	4.1	4.1	3.8	**4.2**
**Gait (*n* = 35)**	2.7	4.9	4.1	4.0	1.7	4.9	5.0	4.9	4.4	4.9	5.0	4.7	4.6	4.6	4.6	4.6	4.9	4.7	**4.4**
**Gait (*n* = 33)**	4.5	4.6	4.5	4.5	4.6	5.0	5.0	5.0	5.0	5.0	5.0	4.9	5.0	5.0	4.7	4.7	4.7	5.0	**4.8**
**Gait (*n* = 32)**	4.1	4.1	3.8	4.0	3.9	4.0	4.0	3.7	3.8	3.8	3.9	3.5	3.2	4.3	3.9	2.6	4.0	3.8	**3.8**
**Gait (*n* = 16)**	3.7	4.3	3.5	3.6	3.9	4.3	4.3	4.5	4.4	4.1	4.8	3.9	4.1	4.4	3.9	3.7	4.0	4.0	**4.1**
**Vaquejada (*n* = 18)**	4.3	4.5	4.2	4.3	4.4	4.4	4.4	4.4	4.4	4.9	5.0	4.4	4.1	4.5	4.4	4.4	4.4	4.3	**4.4**
**Mean of the evaluations of the subdomains**	3.9	4.5	4.1	4.1	4.0	4.5	4.4	4.5	4.4	**4.6**	4.7	4.4	4.2	4.4	4.4	4.2	4.4	4.2	
**Overall averages of the ambience domain—stables**																			**4.3**
(**B**) **General frequency of the levels of quality of well-being and risks according to the sDMs of ambience—stables**																			
**Quality levels of AW and risks**	**sDMs (stables) of the ambience DM**																		**Ambience DM-** **stables**
	**A**	**B**	**C**	**D**	**E**	**F**	**G**	**H**	**I**	**J**	**K**	**L**	**M**	**N**	**O**	**P**	**Q**	**R**	
**Good/excellent** **(>4.0);** **low risk**	62%	100%	62%	75%	50%	100%	100%	87%	75%	87%	87%	75%	75%	87%	75%	75%	100%	75%	**80.4%**
**Fair** **(3.01–3.99);** **medium risk**	25%	0%	38%	25%	38%	0%	0%	13%	25%	13%	13%	25%	25%	0%	25%	13%	0%	25%	**16.8%**
**Terrible/poor** **(<3.0);** **high risk**	13%	0%	0%	0%	12%	0%	0%	0%	0%	0%	0%	0%	0%	13%	0%	12%	0%	0%	**2.7%**

**Notes**: A—Isolation of the technical stable area from the public; B—bearing capacity; C—floor specificity; D—floor drainage; E—floor hygiene; F—functionality; G—specificity; H—accessibility; I—lighting; J—air circulation; K—air quality (presence of odours, dust, and smoke); L—room temperature; M—ambient sound; N—shading/shelter; O—troughs for food; P—drinking fountains; Q—length of stay; R-stable management. *n* represents the number of evaluations per event. The scoring scale ranged from 1 (poor) to 5 (excellent) (Figure 2); sDMs: subdomains; DM: domain; AW: animal welfare.

**Table 5:** Mean results of the evaluations of the ambience domain—competition arenas and their subdomains (*n* = 11) according to the events (**A**) and their quality levels of well-being (**B**).

**Ambience Domain—Competition Arenas**												
**(A) Evaluations of the events according to the different sDMs of the ambience—competition arenas**												
**Events and number of evaluations**	**sDMs (competition arena) of the ambience DM**											**Overall means of the sDMs per event**
	**A**	**B**	**C**	**D**	**E**	**F**	**G**	**H**	**I**	**J**	**K**	
**Vaquejada (*n* = 8)**	4.9	4.8	4.6	4.9	4.8	4.5	4.8	4.9	4.9	4.1	4.7	**4.7**
**Gait (*n* = 8)**	4.7	4.7	4.8	4.9	4.8	4.6	4.6	4.9	4.6	4.5	4.3	**4.7**
**Vaquejada (*n* = 25)**	4.1	4.4	4.1	4.1	4.1	3.6	4.5	4.6	4.4	4.1	3.8	**4.2**
**Gait (*n* = 35)**	4.4	4.7	4.6	4.9	5.0	4.8	4.6	5.0	5.0	4.5	4.6	**4.7**
**Gait (*n* = 33)**	4.8	4.8	4.9	5.0	5.0	5.0	4.9	5.0	4.8	4.9	5.0	**4.9**
**Gait (*n* = 32)**	4.1	4.0	4.1	4.1	4.0	4.0	4.0	4.0	3.3	3.4	3.7	**3.9**
**Gait (*n* = 16)**	4.4	4.4	4.6	4.6	4.6	4.4	4.6	4.8	4.0	4.1	4.3	**4.4**
**Vaquejada (*n* = 18)**	4.6	4.3	4.6	4.4	4.5	4.4	4.5	4.9	4.6	3.9	4.3	**4.5**
**Means of subdomain evaluations**	**4.5**	**4.5**	**4.5**	**4.6**	**4.6**	**4.4**	**4.6**	**4.8**	**4.5**	**4.2**	**4.3**	
**Overall mean of the ambience domain—competition arenas**												**4.50**
**(B) General frequency of the levels of quality of well-being and risks according to the sDMs of the ambience—competition arenas**												
**Quality levels of AW and risks**	**sDMs (competition arenas) of the ambience DM**											**Ambience DM-competition arenas**
	**A**	**B**	**C**	**D**	**E**	**F**	**G**	**H**	**I**	**J**	**K**	
**Good/excellent** **(>4.0);** **low risk**	100%	100%	100%	100%	100%	87%	100%	100%	87%	87%	75%	**94.2%**
**Fair** **(3.01–3.99);** **medium risk**	0%	0%	0%	0%	0%	13%	0%	0%	13%	13%	25%	**5.8%**
**Terrible/poor** **(<3.0);** **high risk**	0%	0%	0%	0%	0%	0%	0%	0%	0%	0%	0%	**0.0%**

**Notes**: A—Floor of the competition site; B—drainage and hygiene; C—perimeter fence; D—functionality; E—specificity; F—accessibility; G—lighting; H—air quality; I—room temperature; J—ambient sound; K—management in the competition environment. *n* represents the number of evaluations per event. The scoring scale ranged from 1 (poor) to 5 (excellent) (Figure 2); sDMs: subdomains; DM: domain; AW: animal welfare.

**Table 6:** Mean results of the evaluations of the nutrition domain and its subdomains (*n* = 11) according to the events (**A**) and their levels of quality of well-being (**B**).

**Nutrition Domain**												
(**A**) **Evaluations of the events according to the different sDMs of nutrition**												
**Events and number of evaluations**	**sDMs of the Nutrition DM**											**Overall means of the sDMs per event**
	**A**	**B**	**C**	**D**	**E**	**F**	**G**	**H**	**I**	**J**	**K**	
**Vaquejada (*n* = 8)**	4.1	4.8	4.5	4.2	4.9	4.6	4.8	4.8	4.8	4.3	4.3	**4.6**
**Gait (*n* = 8)**	4.3	4.1	4.0	4.3	4.4	4.4	4.1	4.3	3.8	4.4	4.7	**4.2**
**Vaquejada (*n* = 25)**	4.1	4.1	4.1	4.2	4.5	4.1	4.4	4.5	4.4	4.1	4.1	**4.3**
**Gait (*n* = 35)**	4.7	4.4	4.5	4.8	4.9	5.0	4.7	4.6	4.9	4.9	5.0	**4.7**
**Gait (*n* = 33)**	4.5	4.7	4.9	4.9	5.0	5.0	5.0	4.8	4.7	5.0	5.0	**4.8**
**Gait (*n* = 32)**	2.8	2.9	3.5	3.2	4.0	3.6	3.9	3.9	3.6	3.9	3.9	**3.5**
**Gait (*n* = 16)**	3.2	3.8	3.4	3.9	4.3	3.9	4.3	4.4	3.5	4.3	4.0	**3.9**
**Vaquejada (*n* = 18)**	4.3	4.4	4.3	4.4	4.6	4.4	4.6	4.5	4.0	4.4	4.3	**4.4**
**Means of subdomain evaluations**	**4.0**	**4.2**	**4.2**	**4.3**	**4.5**	**4.4**	**4.5**	**4.5**	**4.2**	**4.4**	**4.4**	
**Overall mean of the nutrition domain**												**4.30**
(**B**) **General frequency of the levels of quality of well-being and risks according to the sDMs of the nutrition domain**												
**Quality levels of AW and risks**	**sDMs of the nutrition DM**											**Nutrition DM**
	**A**	**B**	**C**	**D**	**E**	**F**	**G**	**H**	**I**	**J**	**K**	
**Good/excellent** **(>4.0);** **low risk**	75%	75%	75%	75%	100%	75%	87%	87%	63%	87%	87%	**80.5%**
**Fair** **(3.01–3.99);** **medium risk**	13%	13%	25%	25%	0%	25%	13%	13%	37%	13%	13%	**17.3%**
**Terrible/poor** **(<3.0);** **high risk**	12%	12%	0%	0%	0%	0%	0%	0%	0%	0%	0%	**2.2%**

**Notes**: A—Water source and storage; B—amount of water; C—water quality; D—accessibility to water; E—accessibility to food; F—food storage; G—frequency of food distribution; H—quantity of forages; I—forage quality; J—amount of concentrates; K—concentrate quality. *n* represents the number of evaluations per event. The scoring scale ranged from 1 (poor) to 5 (excellent) (Figure 2); sDM: subdomains; DM: domain; AW: animal welfare.

**Table 7:** Mean results of the evaluations of the health domain and its subclasses (*n* = 10) according to the events (**A**) and their levels of quality of well-being (**B**).

**Health Domain**											
(**A**) **Evaluations of the events according to the different sDMs of health**											
**Events and number of evaluations**	**sDMs of the Health DM**										**Overall means of the sDMs per event**
	**A**	**B**	**C**	**D**	**E**	**F**	**G**	**H**	**I**	**J**	
**Vaquejada (*n* = 8)**	3.5	4.3	4.8	4.5	4.5	4.5	4.6	4.5	3.5	4.4	**4.30**
**Gait (*n* = 8)**	4.5	4.5	4.5	4.5	4.5	4.8	4.8	4.5	4.0	4.5	**4.50**
**Vaquejada (*n* = 25)**	3.8	3.8	3.6	4.1	4.1	4.1	4.6	4.1	2.5	2.7	**3.75**
**Gait (*n* = 35)**	4.5	3.7	4.6	4.8	4.9	4.6	5.0	4.9	2.8	2.1	**4.20**
**Gait (*n* = 33)**	4.8	4.7	4.6	5.0	5.0	4.7	5.0	5.0	2.1	2.4	**4.33**
**Gait (*n* = 32)**	3.8	3.7	4.2	4.4	4.3	4.8	4.7	3.8	4.0	4.1	**4.18**
**Gait (*n* = 16)**	2.6	2.1	3.0	4.3	4.5	3.8	4.6	4.4	1.4	1.5	**3.21**
**Vaquejada (*n* = 18)**	3.5	5.0	3.0	4.5	4.6	3.7	4.7	4.5	2.4	2.2	**3.81**
**Means of subdomain evaluations**	**3.9**	**4.0**	**4.0**	**4.5**	**4.6**	**4.4**	**4.7**	**4.5**	**2.8**	**3.0**	
**Overall mean of the health domain**											**4.04**
(**B**) **General frequency of the levels of quality of well-being and risks according to the sDMs of Health**											
**Quality levels of AW and risks**	**sDMs of the health DM**										**Health DM**
	**A**	**B**	**C**	**D**	**E**	**F**	**G**	**H**	**I**	**J**	
**Good/excellent** **(>4.0);** **low risk**	37%	50%	63%	100%	100%	75%	100%	88%	25%	38%	**67.6%**
**Regular** **(3.01–3.99);** **medium risk**	50%	37%	37%	0%	0%	25%	0%	13%	13%	0%	**17.5%**
**Terrible/poor** **(<3.0);** **high risk**	13%	13%	0%	0%	0%	0%	0%	0%	62%	62%	**15.0%**

**Notes:** A—Zoosanitary inspection and documentation; B—veterinary care; C—physical integrity of the animals; D—ambulation of the animals; E—body score of the horses; F—absence of signs of disease; G—absence of ectoparasites; H—hydration level; I—veterinary evaluation after the competition; J—evaluation of harnesses and riding equipment. *n* represents the number of evaluations per event. The scoring scale ranged from 1 (poor) to 5 (excellent) (Figure 2); sDMs: subdomains; DM: domain; AW: animal welfare.

**Table 8:** Mean results of the evaluations of the behaviour domain and its subdomains (*n* = 7) according to the event (**A**) and its levels of quality of well-being (**B**).

**Behaviour Domain**								
(**A**) **Evaluations of the events according to the different sDMs**								
**Events and number of evaluations**	**sDMs of the behaviour DM**							**Overall means of the sDMs per event**
	**A**	**B**	**C**	**D**	**E**	**F**	**G**	
**Vaquejada (*n* = 8)**	4.8	4.8	4.6	4.6	4.5	4.8	4.8	**4.7**
**Gait (*n* = 8)**	4.8	4.5	4.5	4.6	4.4	4.5	4.5	**4.5**
**Vaquejada (*n* = 25)**	4.6	4.6	4.6	4.6	4.5	4.6	4.6	**4.5**
**Gait (*n* = 35)**	5.0	5.0	4.7	4.9	4.3	5.0	4.9	**4.8**
**Gait (*n* = 33)**	5.0	5.0	4.6	4.9	5.0	5.0	4.9	**4.9**
**Gait (*n* = 32)**	4.2	3.7	3.9	4.6	4.2	4.3	4.3	**4.1**
**Gait (*n* = 16)**	4.4	4.1	4.5	4.3	4.1	4.5	4.4	**4.3**
**Vaquejada (*n* = 18)**	4.7	4.7	4.7	4.7	4.7	4.7	4.7	**4.7**
**Means of subdomain evaluations**	**4.7**	**4.5**	**4.5**	**4.6**	**4.4**	**4.7**	**4.6**	
**Overall mean of the behaviour domain**								**4.60**
**(B) General frequency of the levels of quality of well-being and risks according to the sDMs of the behaviour DM**								
**Quality levels of AW and risks**	**sDMs of the behaviour DM**							**Behaviour DM**
	**A**	**B**	**C**	**D**	**E**	**F**	**G**	
**Good/excellent** **(>4.0);** **low risk**	100%	87%	87%	100%	100%	100%	100%	**96.3%**
**Regular** **(3.01–3.99);** **medium risk**	0%	13%	13%	0%	0%	0%	0%	**3.6%**
**Terrible/poor** **(<3.0);** **high risk**	0%	0%	0%	0%	0%	0%	0%	**0.0%**

**Notes:** A—Typical behaviour of the species; B—positive interaction between animals of the same species; C—positive interaction with humans; D—absence of stereotypy; E—absence of negative reactions to handling and mounting equipment; F—typical feeding pattern of the species; G—typical resting pattern of the species. *n* represents the number of evaluations per event. The scoring scale ranged from 1 (poor) to 5 (excellent); sDMs: subdomains; DM: domain; AW: animal welfare.

**Table 9:** Mean results of the evaluations of the mental domain and its subdomains (*n* = 7) according to the event (**A**) and its levels of quality of well-being (**B**).

**Mental Domain**												
(**A) Evaluations of the events according to the different sDMs of the mental DM**												
**Events and number of evaluations**	**sDMs of the mental DM**											**Overall means of the sDMs per event**
	**A**	**B**	**C**	**D**	**E**	**F**	**G**	**H**	**I**	**J**	**K**	
**Vaquejada (*n* = 8)**	4.0	4.8	4.8	4.4	4.4	4.8	4.4	4.5	4.6	4.8	4.8	**4.5**
**Gait (*n* = 8)**	4.6	4.8	4.6	4.6	4.6	4.4	4.3	4.0	4.1	4.4	4.5	**4.4**
**Vaquejada (*n* = 25)**	4.6	4.6	4.6	4.6	4.6	4.6	4.6	4.5	4.5	4.6	4.6	**4.5**
**Gait (*n* = 35)**	4.4	4.7	4.8	4.9	4.9	5.0	4.8	4.8	4.6	5.0	4.9	**4.8**
**Gait (*n* = 33)**	4.9	4.9	4.8	5.0	5.0	4.9	5.0	5.0	4.7	5.0	5.0	**4.9**
**Gait (*n* = 32)**	4.4	4.2	4.0	4.7	4.6	4.5	4.1	4.1	4.2	4.4	4.5	**4.3**
**Gait (*n* = 16)**	4.5	4.5	4.4	4.6	4.4	4.6	4.1	4.4	4.1	4.3	4.3	**4.4**
**Vaquejada (*n* = 18)**	4.7	4.7	4.7	4.7	4.7	4.7	4.7	4.7	4.7	4.7	4.7	**4.7**
**Overall means of subdomain evaluations**	**4.5**	**4.6**	**4.6**	**4.7**	**4.7**	**4.7**	**4.5**	**4.5**	**4.5**	**4.6**	**4.7**	
**Overall mean of the mental domain**												**4.60**
**(B) General frequency of the levels of quality of well-being and risks according to the sDMs of the mental DM**												
**Quality levels of AW and risks**	**sDMs of the mental DM**											**Mental DM**
	**A**	**B**	**C**	**D**	**E**	**F**	**G**	**H**	**I**	**J**	**K**	
**Good/excellent** **(>4.0);** **low risk**	100%	100%	100%	100%	100%	100%	100%	100%	100%	100%	100%	**100.0%**
**Regular** **(3.01–3.99);** **medium risk**	0%	0%	0%	0%	0%	0%	0%	0%	0%	0%	0%	**0.0%**
**Terrible/poor** **(<3.0);** **high risk**	0%	0%	0%	0%	0%	0%	0%	0%	0%	0%	0%	**0.0%**

**Notes:** A—Absence of fear; B—maintenance of attention; C—absence of leisure; D—absence of agitation; E—absence of anxiety; F—depression; G—joy/happiness; H—engagement in exploring the environment; I—absence of fatigue (hyporeactivity); J—gregarious behaviour; K—food satiety. *n* represents the number of evaluations per event. The scoring scale ranged from 1 (poor) to 5 (excellent) (Figure 2); sDMs: subdomains; DM: domain; AW: animal welfare.

## Data Availability

The data presented in this study are available upon request from the corresponding author.
